# SARS-CoV-2 Antibodies Are Persisting in Saliva for More Than 15 Months After Infection and Become Strongly Boosted After Vaccination

**DOI:** 10.3389/fimmu.2021.798859

**Published:** 2021-12-09

**Authors:** Yudi T. Pinilla, Constanze Heinzel, Lena-Fabienne Caminada, Douglas Consolaro, Meral Esen, Peter G. Kremsner, Jana Held, Andrea Kreidenweiss, Rolf Fendel

**Affiliations:** ^1^ Institute of Tropical Medicine, University Hospital Tübingen, Tübingen, Germany; ^2^ German Center for Infection Research (DZIF), Partner Site Tübingen, Tübingen, Germany; ^3^ Centre de Recherches Médicales de Lambaréné, Lambaréné, Gabon; ^4^ Cluster of Excellence: Exzellenzcluster (EXC) 2124: Controlling Microbes to Fight Infection, Tübingen, Germany

**Keywords:** SARS-CoV-2, COVID-19, antibody, saliva, mucosa, convalescent, vaccination, neutralization

## Abstract

SARS-CoV-2 antibodies in saliva serve as first line of defense against the virus. They are present in the mucosa, more precisely in saliva, after a recovered infection and also following vaccination. We report here the antibody persistence in plasma and in saliva up to 15 months after mild COVID-19. The IgG antibody response was measured every two months in 72 participants using an established and validated in-house ELISA assay. In addition, the virus inhibitory activity of plasma antibodies was assessed in a surrogate virus neutralization test before and after vaccination. SARS-CoV-2-specific antibody concentrations remained stable in plasma and saliva and the response was strongly boosted after one dose COVID-19 vaccination.

## Introduction

The COVID-19 pandemic has now been ongoing since December 2019 and has received major social, political, and scientific attention from the beginning. Both SARS-CoV-2 infection and COVID-19 vaccination elicit an immune response that largely protects from (re)infection and severe disease (1). Although immune correlates of protection have not yet been pinned down, antibody-mediated immunity is a critical constituent (2, 3). The SARS-CoV-2 specific humoral immune response characteristics, including magnitude, longevity and function have been - and still are - classically studied for serum immunoglobulins. So far, little attention has been paid to the presence of SARS-CoV-2 antibodies in the mucosa of the upper respiratory tract, the primary entry route for the virus (4). An established model for the investigation of the role of mucosal immunity against oral infection is the murine cytomegalovirus (5), in which a specific vaccination elicits high antibody titers in salivary glands that are leading to the protection from an otherwise deadly challenge (6). This mechanism has also been proposed for other respiratory viruses such as the influenza A virus (7, 8). Evidence is accumulating that SARS-CoV-2 spike protein reactive antibodies in respiratory mucosa can also control virus replication (9). In a nonhuman primate infection model, it was shown that mRNA-1273 (Moderna) vaccinated animals with higher spike protein-specific IgG in the mucosa were less likely to be infected after challenge, and naïve hamsters were protected by passive transfer of vaccine-induced IgG (10). SARS-CoV-2 antibodies are present in the mucosa, more precisely in saliva, after a recovered infection and the following vaccination for at least 9 months (11–14).

To shed light on the association between antibodies in plasma and in mucosa, we compared the kinetics of the SARS-CoV-2 spike protein IgG concentrations over a 15-month follow-up period after recovery from mild COVID-19 disease. We further investigated if the antibody response deriving from the SARS-CoV-2 infection is boosted following one dose of a commercially available COVID-19 vaccine that could be able to control SARS-CoV-2 infection and transmission.

## Methods

### Study Design

Between May and October 2020 adults who had a SARS-CoV-2 infection had been externally diagnosed by qPCR (n = 40) or ELISA (n = 32) and documented by a medical certificate were included in the study. The reported date of symptom onset (PSO) served as a surrogate date for SARS-CoV-2 infection. Duration of disease and symptoms were collected *via* a questionnaire.

### Sample Collection and Processing

Sample processing and storage was done as published recently (12). Briefly, saliva was collected by spitting into a sterile container (3ml) (Multi-purpose containers 30ml Greiner Bio-One ref. 201150) and kept at 4°C with a maximum of 6 hours until sample processing. The tubes were centrifuged at 2500 rpm for 6 min and the supernatant was inactivated using tri-n-butyl phosphate (TnBP) 0.3% and Triton X-100 1% at final concentrations. Samples were stored at -20°C for the further analysis. Peripheral blood was collected using 9 ml lithium heparin monovettes. Plasma was isolated and immediately collected by centrifugation at 1400 rpm for 10 min and stored at -20°C until use.

### ELISA for SARS-CoV-2 IgG Detection in Saliva and Plasma

IgG reactive to SARS-CoV-2 RBD were measured by a meticulously established and validated in-house ELISA (12, 13) for IgG detection in saliva as well as by an in-house ELISA specifically established to analyze plasma antibodies (see **Supplementary Material**). Briefly, the SARS-CoV-2 RBD antigen was diluted in 1x PBS to a final concentration of 2 μg/ml and added per well to high binding plates. After overnight incubation, the wells were washed and blocked with The Blocking Solution for 2 hours at room temperature (RT) on a microplate shaker (700 rpm). Subsequent washing steps were repeated. Saliva and control samples were serially diluted from 1:3 up to 1:19,683 using The Blocking Solution. 100 μl sample dilution was added per well and incubated for 1 hour (RT, 700 rpm). IgG antibody presence was detected by 1:20,000 diluted biotinylated anti-human IgG and 1:1,000 Avidin-HRP. For visualization, 100 µl TMB substrate solution was added, and the reaction was stopped using 1 M HCl. The plate was read at 450 nm and 620 nm with a microplate reader (CLARIOstar, BMG LABTECH). Cut-off for salivary SARS-CoV-2 IgG positivity was previously set to 6.3 ng/ml (12).

The SARS-CoV-2 IgG detection in plasma was performed using an established in-house assay following similar parameters used in the saliva ELISA assay, with some exceptions: Plasma was diluted 1:100 up to 1:7,812,500 dilution in The Blocking solution (1 to 5 dilution row) and for IgG detection an HRP coupled anti-human IgG was used (ref. 109-036-097, Jackson Immuno Research Laboratories). The detection antibody was diluted 1:5,000 in 1x ROTI Block buffer and incubated for 30 min (RT, 700 rpm). The IgG concentration is presented in µg/ml.and the cut-of value was set to 4.0 µg/ml (12). For confirmation, plasma samples were re-analyzed by the commercial, CE certified SARS-CoV-2 IgG ELISA (EUROIMMUN) detecting antibodies binding to SARS-CoV-2 Spike protein domain S1 and the assays were performed following the manufacturer’s instructions.

### Surrogated Virus Neutralization Assay

A surrogate virus neutralization test (SARS-CoV-2 NeutraLISA, ref. EI2606-9601-4 kindly provided by EUROIMMUN) was performed following manufacturer’s instruction to estimate the inhibitory activity of SARS-CoV-2 IgG in plasma in a non-BSL-3 set-up (see **Supplementary Material** for detailed description of the assay procedures).

### Data Analysis

RStudio (Version 1.2.5001), running R (version 4.0.4.) and Graph Pad Prism (Version 9.1) were used for descriptive statistical analyses. The data was analyzed at 95% confidence interval (p<0.05). Linear regression modelling was performed to assess change of IgG concentrations over time, and local polynomial regression was used to model the kinetics of antibody levels before and after vaccination using the stats package (version 3.6.2) in R. Pairwise Pearson’s product-moment correlation coefficient was applied to assess the correlation of IgG concentration in saliva and in plasma as well as between % neutralization and plasma IgG concentrations. Non parametric Wilcoxon rank sum paired test was performed to check for significant difference of IgG concentrations or of % neutralization before and after vaccination.

For all statistical analyses, significance level (alpha) was set to 0.05.

## Results

Between May and October 2020, 72 adults (male/female; n = 25/47, median (range) age in years = 29 (19–75)) who had suffered from mild COVID-19 (all participants not hospitalized, loss of smell and taste: n = 50) were enrolled to monitor and longitudinally compare the kinetics of SARS-CoV-2 reactive IgG in saliva and in plasma after convalescence. Additionally, the virus inhibitory activity of plasma antibodies in a surrogate virus neutralization test was assessed.

Approximately 4 months (median: 132 days, range: 42-171 days) post COVID-19 symptom onset (PSO), first sampling was done, and 89% (64/72) participants were positive for SARS-CoV-2 RBD specific IgG in plasma as analyzed by the established in-house ELISA and confirmed by commercial EUROIMMUN ELISA (58/72 were IgG positive and 5/72 were IgG intermediate) (**Supplementary Figures 1A, B**). Participants were followed for 5 additional bimonthly timepoints, at which saliva and plasma were collected, covering a total period of in average 15 months after SARS-CoV-2 infection (median PSO: 447 days, range: 367–497 days). During the follow-up period, none reported a re-infection, but 58% (42/72) participants received COVID-19 vaccination (22 Corminaty/BioNTech, 7 Spikevax/Moderna, 13 Vaxzevria/AstraZeneca) at any time between 235 and 409 days PSO.

**Figure 1:**
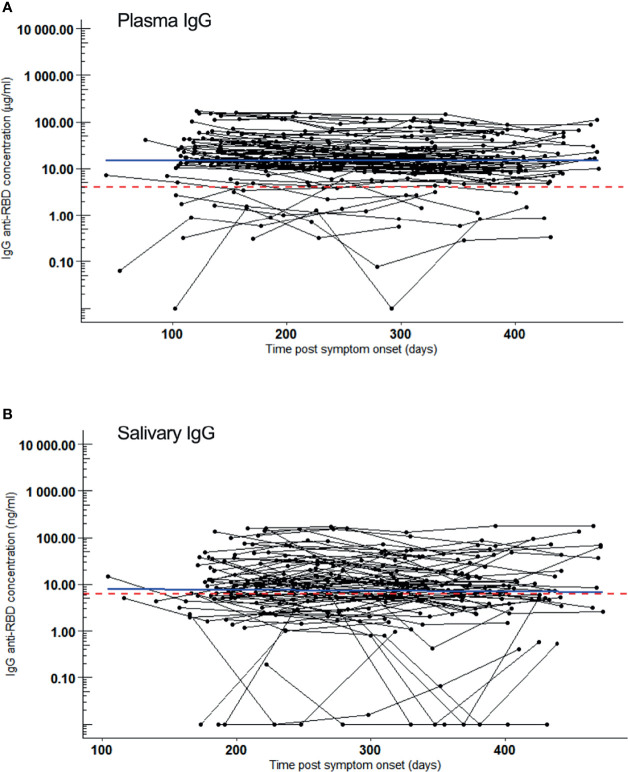
SARS-CoV-2 RBD reactive IgG over a 15-month follow-up period. Samples were collected 5 times, bimonthly from 72 convalesced volunteers who had mild COVID-19. IgG kinetics are shown per individual (black line). Sampling time points (black dots) are given as days post symptom onset (PSO). Blue line: regression line. Red dotted line: Cut-off for positivity. **(A)** IgG (µg/ml) kinetics in plasma after COVID-19 convalescence. Slope of linear regression = -0.02, p = 0.2. **(B)** IgG (ng/ml) kinetics in saliva after COVID-19 convalescence. Slope of linear regression = 0.009, p = 0.7.

Half a year after infection (median PSO: 196 days, range: 104-226 days), SARS-CoV-2 IgG were present in plasma of 89% (64/72) of volunteers or in saliva of 72% (46/72) (all saliva IgG positives were also positive for plasma IgG). At the end of the follow-up period, either 15 months PSO or at the last follow-up before vaccination, still 98% (63/64) and 80% (37/46) had SARS-CoV-2 reactive IgG in plasma and in saliva, respectively. IgG concentrations in plasma (**Figure 1A**) and in saliva (**Figure 1B**) remained constant during this follow-up period of up to 15 months (flat regression lines). IgG concentrations in paired saliva and plasma samples were highly correlating (Pearson’s product-moment correlation r = 0.73, [95% confidence interval (CI): 0.67-0.78)] (**Supplementary Figure 2**). Interstingly, the median IgG concentrations in saliva were 1,206-fold (95% CI: 1,062-fold to 1,493-fold) lower than in plasma.

**Figure 2:**
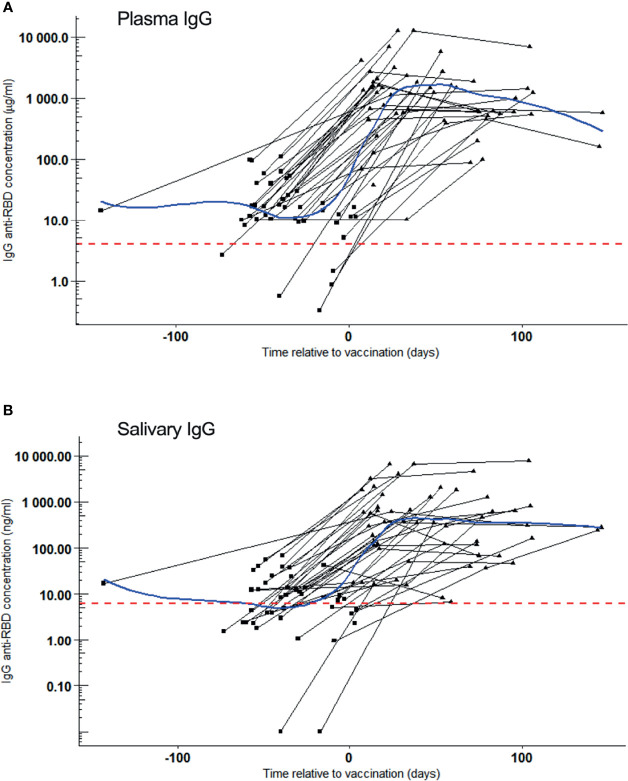
SARS-CoV-2 RBD reactive IgG after vaccination. Samples were collected of whom 42 became vaccinated during the study. IgG kinetics are shown per individual (black line). Sampling time points (black dots) are given as days post symptom onset (PSO). Blue line: Local Polynomial Regression. Red dotted line: Cut-off for positivity. **(A)** Plasma IgG (µg/ml) concentration per convalesced volunteer before (square) and after (triangle) one dose of COVID-19 vaccination. Day 0 represents the reported day of vaccination. Local Polynomial Regression, residual standard error: 1979. **(B)** Salivary IgG (ng/ml) concentration per convalescent volunteer before (square) and after (triangle) one dose of COVID-19 vaccination. Day 0 represents the reported day of vaccination. Local Polynomial Regression, residual standard error: 1354.

COVID-19 vaccination induced a remarkable and significant increase of SARS-CoV-2 RBD reactive IgG concentrations - both in plasma and in saliva of COVID-19 convalescent volunteers (**Figures 2A, B**). IgG concentrations increased 72-fold in plasma (p = 7.2x10^-12^) and 46-fold in saliva (p = 6.9x10^-11^) within one month (median: 26 days) after first vaccination when compared to those before vaccination (median: -39 days), reaching median concentrations of 1458 µg/ml and 626 ng/ml, respectively. Volunteers negative for SARS-CoV-2 IgG in plasma or saliva before vaccination also increased significantly in antibody levels in plasma and saliva (median IgG plasma 449 µg/ml and 137 ng/ml) after vaccination. Although, these concentrations were remarkably lower to those reached by volunteers positive for IgG prior to vaccination (3.3 fold for plasma IgG and 4.6 fold for salivary IgG), this difference did not reach statistical significance (p = 0.21 and p = 0.10 for plasma and saliva, respectively).

**Figure 3:**
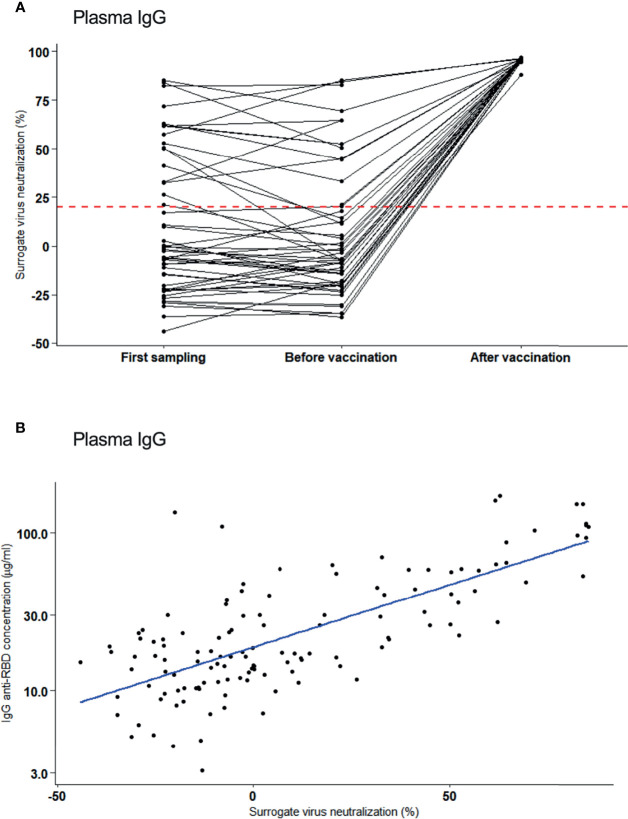
Neutralizing activity of plasma antibodies to SARS-CoV-2. **(A)** Inhibitory activity (%) (surrogate for virus neutralization) of plasma IgG assessed at first sampling (4 months after infection), before vaccination and after vaccination. Neutralization activity are shown per individual (black line). Red dotted line: Cut-off for positivity. **(B)** Correlation of neutralization activity (%) and plasma IgG concentration after one vaccination (Pearson correlation, r=0.70).

To assess the plasma for virus neutralizing antibodies, an ACE2 receptor/antibody competition assay was done as surrogate measure for virus neutralization of those individuals with plasma IgG. At the first sampling (4 months PSO), 34% (17/50) individuals had neutralizing antibodies and this fraction did not largely change until 8.5 months later [median: 254 days, range 111 – 334 days; 27% (14/50)]. Of this cohort, 33 became vaccinated 2 months later (median: 70 days, range: 55 – 167 days) and 100% (33/33) attained high neutralization activity (**Figure 3A**). Neutralization activity in plasma of vaccinated participants was significantly different from those who remained unvaccinated (median inhibition of 96% versus 10%, respectively, p = 3.3x10^-8^). Notably, SARS-CoV-2 RBD IgG concentrations in plasma correlated positively with neutralization activity [Pearson correlation, r=0.70 (**Figure 3B**)].

## Discussion

SARS-CoV-2 immunity in the respiratory mucosa, particularly in saliva, includes locally secreted dimeric IgA as well as IgG that comes largely from the systemic immune response *via* gingival transduction in the gums (15). Correlating IgG plasma and saliva levels from recovered COVID-19 patients have been reported to persist for at least 3 months (15–17). Our study provides evidence of long-term and constant SARS-CoV-2 RBD IgG levels for most COVID-19 convalescent individuals not only in plasma but - to our knowledge - for more than one year in saliva. In the absence of a defined correlate of protection, SARS-CoV2 RBD IgG levels serve as a proxy for monitoring immune response. Here, one vaccination dose strongly boosted IgG levels not only in plasma but also in saliva. SARS-CoV-2 is transmitted orally and virus spike protein binding to the host angiotensin converting enzyme 2 (ACE2) of epithelial cells in the respiratory pathways permits virus infection and replication. If mucosal IgG elicited by a past SARS-CoV-2 infection and/or by vaccination can prevent (re)infection, replication and transmission, e.g. by interfering with spike protein binding, still needs to identified and quantified (9). Encouragingly, supporting findings come from a recent study in which vaccinated non-human primates with strong mucosal antibody responses were protected from an infection after SARS-CoV-2 challenge, revealing for the first time antibody concentration as a direct mechanistic correlate of protection (10). To gain functional insights into SARS-CoV-2 RBD IgG, a surrogate virus neutralization test was done, but for technical reasons, this was unfortunately only possible for plasma IgG due to low IgG levels in saliva. Remarkably, spike protein binding to ACE2 was only moderately inhibited by IgG in COVID-19 convalescent donors and was strongly increased after already one dose of any of the given vaccine. Future investigations should address the cross-neutralization against circulating SARS-CoV-2 variants of concern, e.g. the currently circulating Delta B.1.617.2 variant. In addition, assays capable of quantifying IgA-levels in saliva are needed, as these might also contribute to protection against SARS-CoV-2 infection.

In summary, evidence towards a role of mucosal antibodies in prevention of SARS-CoV-2 infection is accumulating.

## Data Availability Statement

The original contributions presented in the study are included in the article and the **Supplementary Material**. Further inquiries can be directed to the corresponding author.

## Ethics Statement

The studies involving human participants were reviewed and approved by the Ethics Committee of the University Hospital Tübingen (B312/2020BO1 and 247/2020BO1) and registered at Clinicaltrials.gov (NCT04581889) (18). All participants gave written informed consent to participate in the study.

## Author Contributions

AK, RF, JH, ME, and PK conceived and designed the study. RF and CH designed and conducted all experiments. YP and L-FC did the sample collection and sample processing. YP supervised the study conduct. CH, YP, JH, RF, and AK verified and analysed the data. JH, RF, AK, and DC did the statistical analysis. AK, CH, YP, and RF wrote the first manuscript draft. AK, RF, and JH revised and finalized the manuscript. All authors reviewed and approved the contents of the manuscript. All authors confirm that they had full access to all the data in the study and accept responsibility to submit for publication.

## Funding

The project was supported by the University Hospital Tübingen, Germany. We acknowledge support by the OpenAccess Publishing Fund of the University of Tübingen.

## Conflict of Interest

The authors declare that the research was conducted in the absence of any commercial or financial relationships that could be construed as a potential conflict of interest.

## Publisher’s Note

All claims expressed in this article are solely those of the authors and do not necessarily represent those of their affiliated organizations, or those of the publisher, the editors and the reviewers. Any product that may be evaluated in this article, or claim that may be made by its manufacturer, is not guaranteed or endorsed by the publisher.
