# Supplementary material for: SARS-CoV-2 Antibodies Are Persisting in Saliva for More Than 15 Months After Infection and Become Strongly Boosted After Vaccination
Source: Front Immunol. 2021 Dec 9;12:798859. doi: 10.3389/fimmu.2021.798859 (PMC8695841; doi:10.3389/fimmu.2021.798859)
Supplement: Supplementary file 1 [file DataSheet_1.docx]

**Supplementary Material**

**Methods**

*Antigen expression*

The pCAGGS plasmid encoding for the SARS-CoV-2 receptor-binding domain (RBD) protein was kindly provided by Florian Krammer (Icahn School of Medicine at Mount Sinai, New York) (1). The RBD sequence encodes for amino acids R319 to F541 of the spike protein plus a C-terminal His tag. Recombinant RBD protein has a molecular weight of 27.5 kDa (without glycosylation). Human embryonic kidney Expi293F™ Cells (HEK cells, Thermo Fisher; ref. A14528) cells were transfected with pCAGGS plasmid using ExpiFectamine™ 293 Transfection Kit (Gibco™; ref. A14525). Transfection and supernatant harvest were performed according to the manufacturer's manual. The supernatant was purified by an ÄKTA chromatography system using a HisTrap HP 5 ml column (GE Healthcare, ref. 17524802). Protein size and quality control of the recombinant RBD protein were performed by SDS-Page and Western blot analysis. RBD protein concentration was diluted to 2 mg/ml in 1x PBS supplemented with protease inhibitors (Roche; ref. 11697498001).

*ELISA for SARS-CoV-2 IgG detection in* *saliva*

To measure SARS-CoV-2 RBD specific IgG antibody concentrations in saliva, a recently established and validated ELISA (the saliva ELISA) was used (2). First, the SARS-CoV-2 RBD antigen was diluted in 1x PBS to a final concentration of 2 μg/ml and 50 μl were added per well to coat Costar 96 well microtiter high binding plates (ref. 3590, Corning). After overnight incubation at 4°C, the wells were washed once with 1x PBS and blocked with 200 µl of The Blocking Solution (Condor Bioscience GmbH) for 2 hours at room temperature (RT) on a microplate shaker (700 rpm). Subsequent washing steps were repeated 3x with PBS/0.1% Tween20. Saliva and control samples were serially diluted from 1:3 up to 1:19683 using The Blocking Solution. 100 μl sample dilution was added per well and incubated for 1 hour (RT, 700 rpm). IgG antibody presence was detected by 1:20,000 diluted biotinylated anti-human IgG (ref. 109-065-008, Jackson Immuno Research Laboratories) and 1:1,000 Avidin-HRP (Biolegend ref. 405103). Both reagents were dissolved in 1x ROTI Block buffer (Roth) and incubated for 1 hour and 30 min, respectively. For visualization, 100 µl TMB substrate solution was added, and the reaction was stopped using 50 µl 1 M HCl. The plate was read at 450 nm and 620 nm with a microplate reader (CLARIOstar, BMG LABTECH). Data is given as concentration in ng/ml and was estimated by a respective dilution series of highly pure human IgG, which was precoated on separate wells on the same plates (ref. 31154, ThermoFisher).

*ELISAs for SARS-CoV-2 IgG detection in* *plasma*

Two ELISA tests were performed for SARS-CoV-2 IgG detection in plasma: an in-house SARS-CoV-2 RBD ELISA and the commercial and CE-certified EUROIMMUN SARS-CoV-2 IgG ELISA (EI 2606-9601 G). The in-house ELISA procedure were similar to these of the saliva ELISA with the exceptions of sample dilution and the IgG detection antibody. Plasma was diluted 1:100 up to 1:7,812,500 dilution in The Blocking solution (1 to 5 dilution row) and for IgG detection an HRP coupled anti-human IgG was used (ref. 109-036-097, Jackson Immuno Research Laboratories). The detection antibody was diluted 1:5,000 in 1x ROTI Block buffer and incubated for 30 min (RT, 700 rpm). The IgG concentration is presented in µg/ml.

Plasma samples were analysed using the EUROIMMUN SARS-CoV-2 IgG ELISAs kit, detecting IgG binding to the SARS-CoV-2 Spike protein domain S1. Assays were performed following the manufacturer’s instructions. Plasma specimens were diluted in a provided sample buffer, added to antigen-coated microtiter wells, and then incubated at 37°C for 1 hour. Plates were washed and a conjugated solution was added and incubated at 37°C for 30 min. After a second wash step, the substrate solution was applied and incubated at RT for 30 min. Finally, 0.5 M sulfuric acid stop solution was added and absorbance of the sample wells was measured immediately at 450 nm and 630 nm using the CLARIOstar microplate reader (BMG). Output reports generated with optical density (O.D.) at 630 nm were subtracted from O.D. at 450 nm. The data were analysed as recommended by the manufacturer and results were reported as a ratio. Following EUROIMMUN specifications the cut-off for SARS-CoV-2 IgG positivity is set at ≥ 1.1 ratio, intermediate antibody concentration is at 0.8 to 1.1 ratio, and negative is defined as < 0.8 ratio.

*Surrogated virus neutralization assay*

SARS-CoV-2 neutralization assays using plasma from COVID-19 convalsecent participants who became COVID-19 vaccinated were performed following manufacturer’s instructions of the SARS-CoV-2 NeutraLISA kit (EI2606-9601-4), kindly provided by EUROIMMUN Medizinische Labordiagnostika AG. The kit contains microplate strips coated with recombinant S1 protein of SARS-CoV-2. Briefly, samples were diluted (1:5 to 1:1,250) with a provided buffer containing biotinylated ACE2 and incubated simultaneously on the plate. Following incubation and washing, bound-ACE2 was detected by Streptavidin-peroxidase. TMB was used to elicit the color reaction and the reaction was stopped with 0.5 M sulfuric acid. Results were calculated by the ratio of the extinction of the sample divided by the extinction of the blank calibrator and given as % inhibition. In addition, WHO standard 20/136 (NIBSC) was used to quantify % inhibition of ACE2-binding to S1 protein mediated by plasma sample (SARS-CoV-2 reactive antibodies). As final readout, % neutralizing antibody activity was estimated by a 4 parameter logistic regression model using GraphPad prism v. 9.0.2. An inhibition of 20% is defined as positive as per manufacturer´s specification.


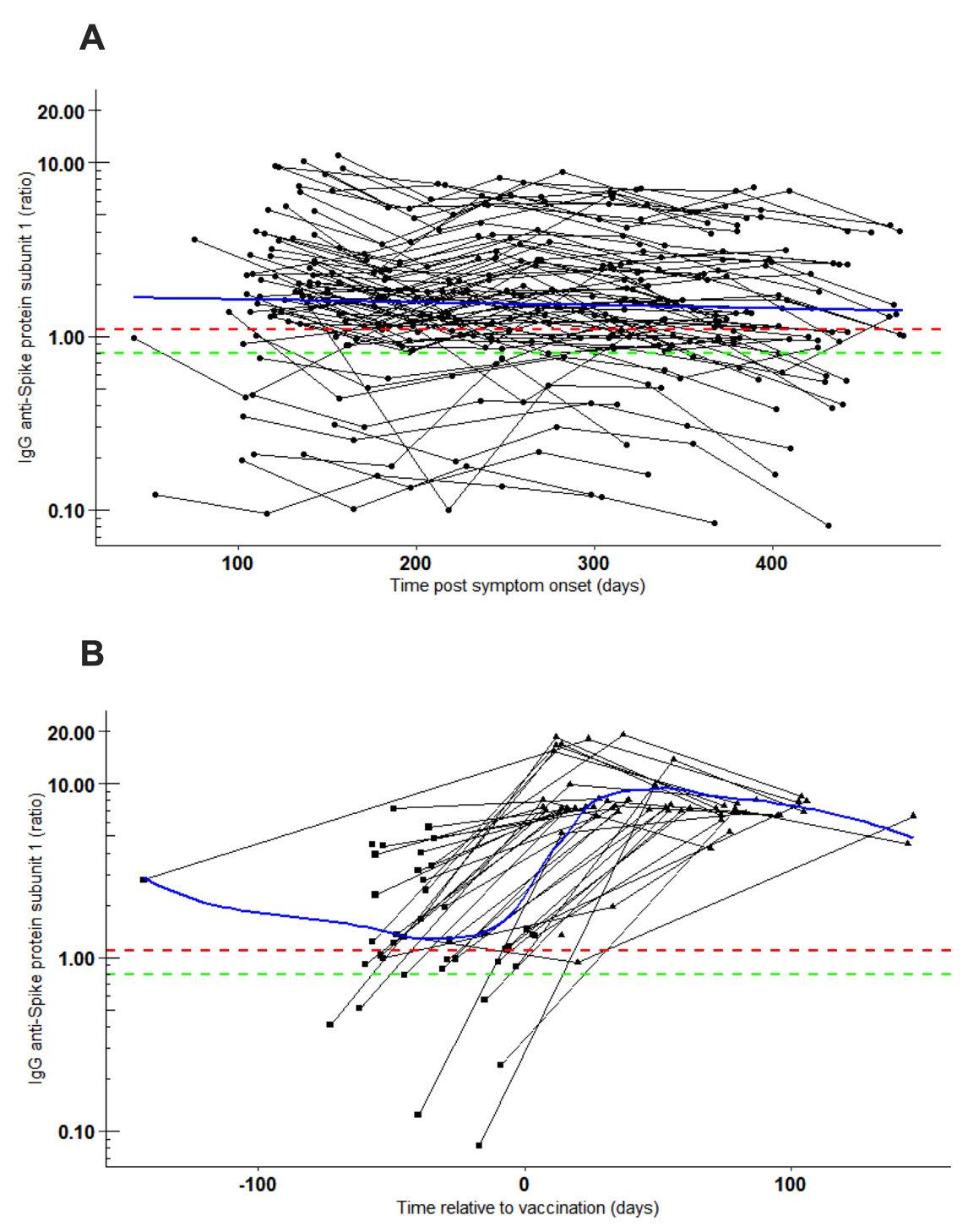


**Supplementary Figure 1.** SARS-CoV-2-Spike protein domain S1 reactive IgG in plasma over a 15-month follow-up period and assessed by commercial EUROIMMUN SARS-CoV-2 IgG ELISAs kit. Samples were collected 5 times, bimonthly from 72 convalesced volunteers who had mild COVID-19 (A) IgG kinetics are shown per individual (black line). Sampling time points (black dots) are given as days post symptom onset (PSO). Blue line: regression line. Red dotted line: Cut-off for positivity, green dotted line: Cut-off for negativity. (A) IgG anti-Spike protein subunit 1 (ratio) kinetics in plasma after COVID-19 convalescence. Slope of linear regression = -0.0015, p = 0.2. (B) Plasma IgG (ratio) concentration per convalescent volunteer before (square) and after (triangle) one dose of COVID-19 vaccination of whom 42 became vaccinated during the study. Day 0 represents the reported day of vaccination. Local Polynomial Regression (residual standard error: 3397).


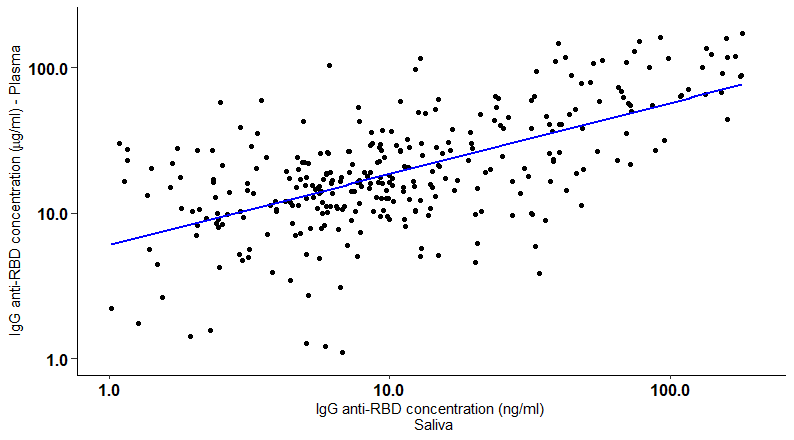


**Supplementary Figure 2.** Correlation between IgG anti-RBD concentration in plasma vs saliva. Data shows a positive correlation r=0.73, (95% CI: 0.67-0.78)

**References**

1. Amanat F, Stadlbauer D, Strohmeier S, Nguyen THO, Chromikova V, McMahon M, et al. A serological assay to detect SARS-CoV-2 seroconversion in humans. Nat Med. 2020;26(7):1033-6.

2. Heinzel C, Pinilla YT, Elsner K, Friessinger E, Mordmüller B, Kremsner PG, et al. Non-Invasive Antibody Assessment in Saliva to Determine SARS-CoV-2 Exposure in Young Children. Frontiers in immunology. 2021;12.
